# Supplementary figures and images for: Evaluation of the United States COVID-19 vaccine allocation strategy
Source: PLoS One. 2021 Nov 17;16(11):e0259700. doi: 10.1371/journal.pone.0259700 (PMC8598051; doi:10.1371/journal.pone.0259700)

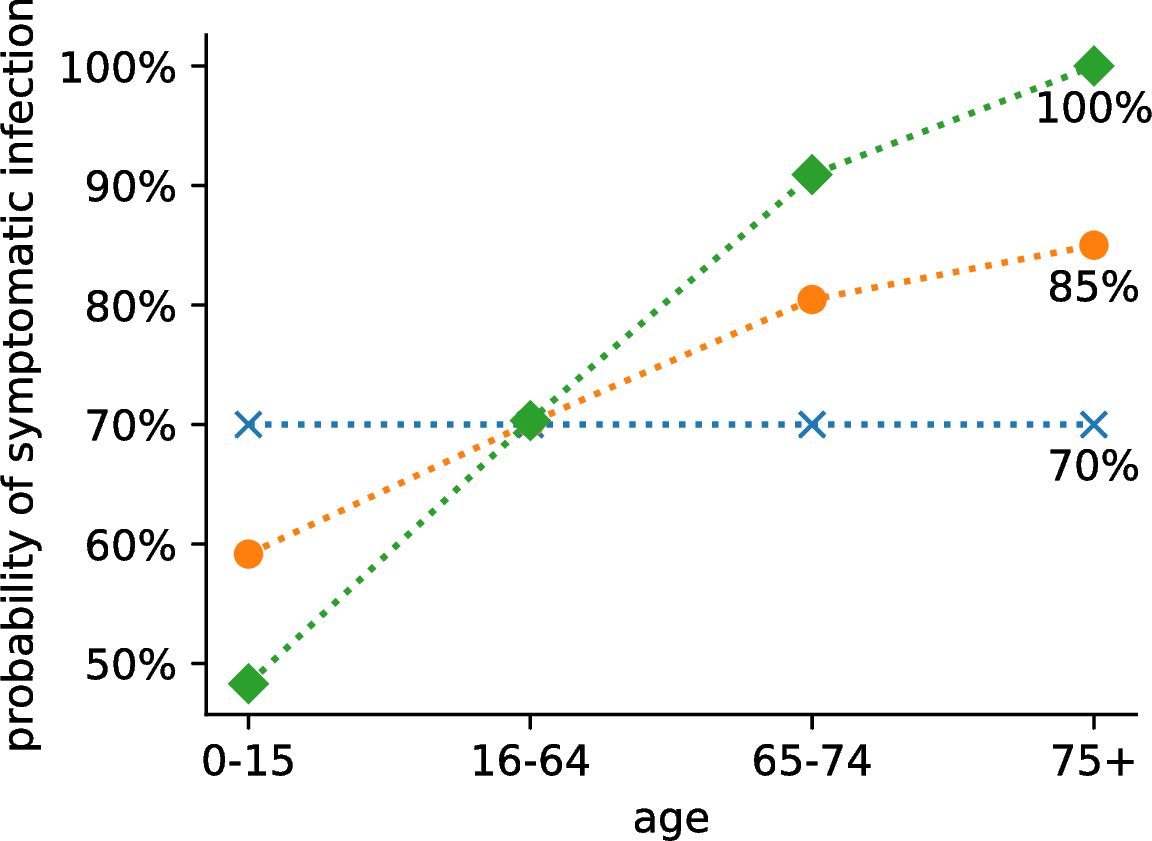

Supplement: S1 Fig — The probability of symptomatic infection is shown for the different age groups (x-axis) and different scenarios (colors). The average probability of symptomatic infection is 70% in each scenario. This probability increases linearly with year of age up to a fixed value of 70% (blue x), 85% (orange circles; default), 100% (green diamonds) for the age group 75+. (TIF) [file pone.0259700.s001.tif]

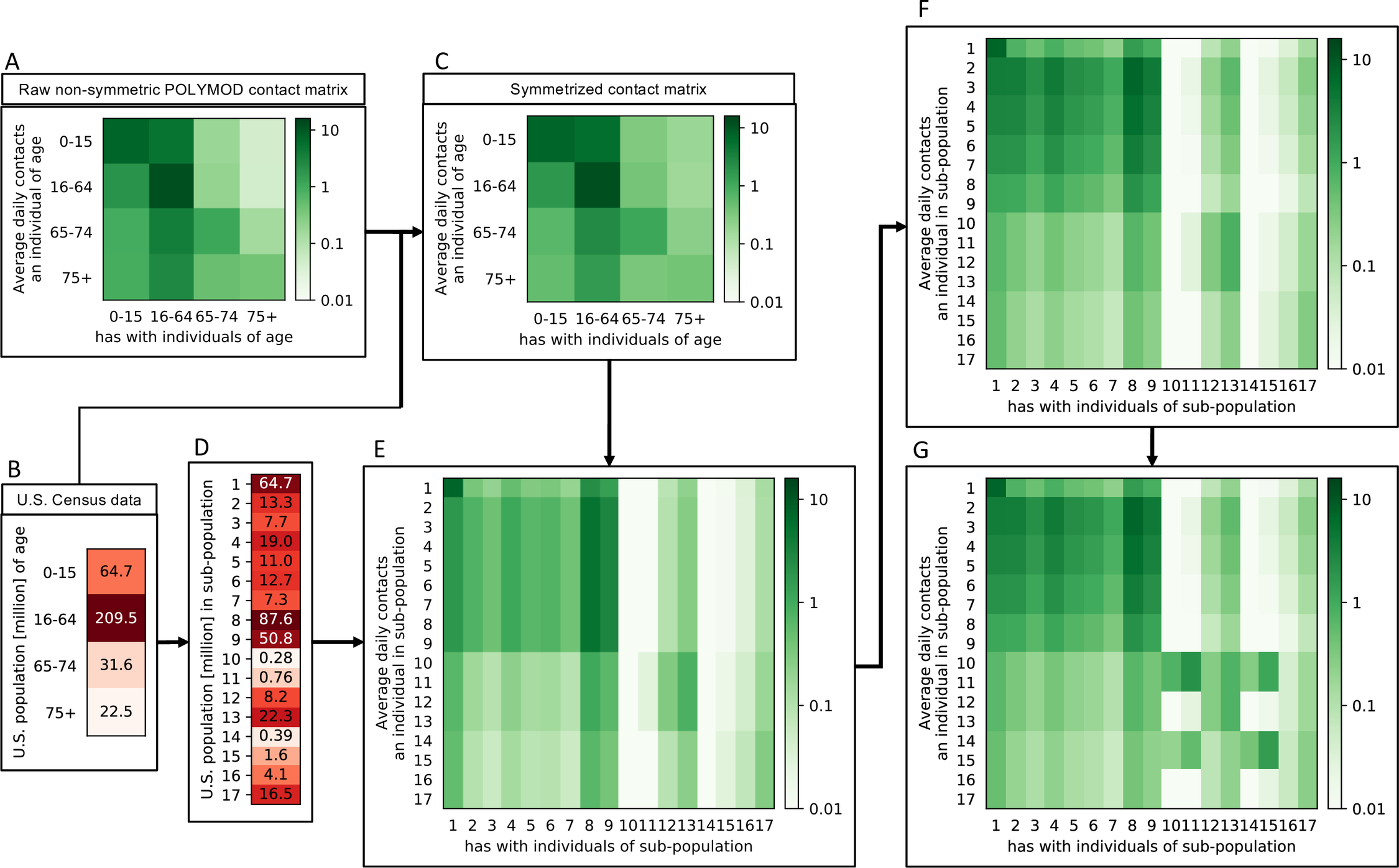

Supplement: S2 Fig — (A) The original 4 × 4 U.S. age-age contact matrix inferred from survey data [20, 21] was transformed, using (B) U.S. census data, into (C) a symmetric 4 × 4 contact matrix [45]. Using (D) information on the number of individuals within each of the 17 sub-populations used in this study (characteristics defined in Table 2), the symmetric 4 × 4 contact matrix was expanded into (E) a 17 × 17 contact matrix. Some jobs require more physical contact than others. Inclusion of the average contact rates per job type yielded (F) an adapted contact matrix. Similarly, elderly individuals in congested living conditions have more contacts than their peers and all these increased contacts were assumed to occur within the congested living environment, which yielded (G) the final contact matrix used in this study. (TIF) [file pone.0259700.s002.tif]

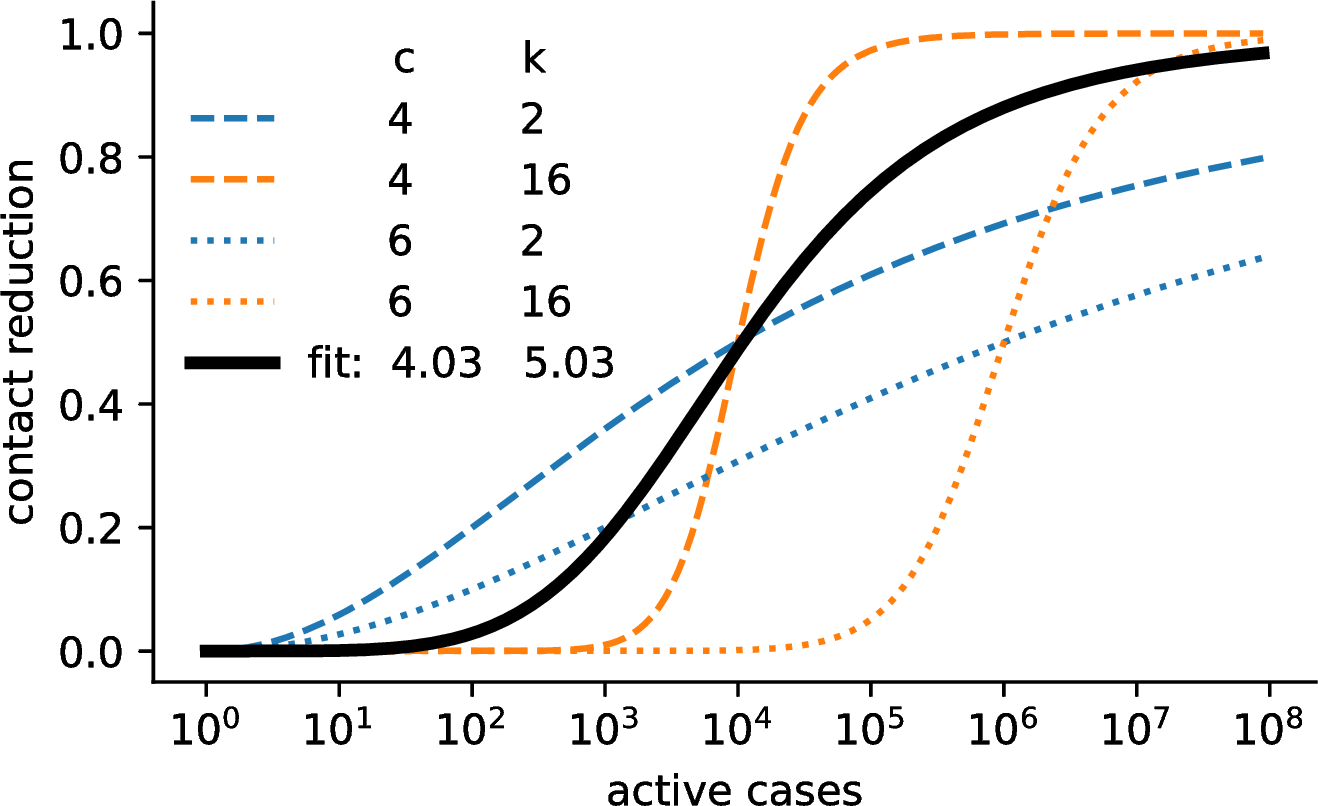

Supplement: S3 Fig — A variable contact reduction (Hill function) accounts for changes in the population-wide activity level based on the severity (i.e., the number of active cases) of the epidemic in the United States. The shape of the case-dependent contact reduction used in the base model (black line) is shown along with the shapes of the most extreme parameter choices allowed in the genetic algorithm (dashed lines). (TIF) [file pone.0259700.s003.tif]

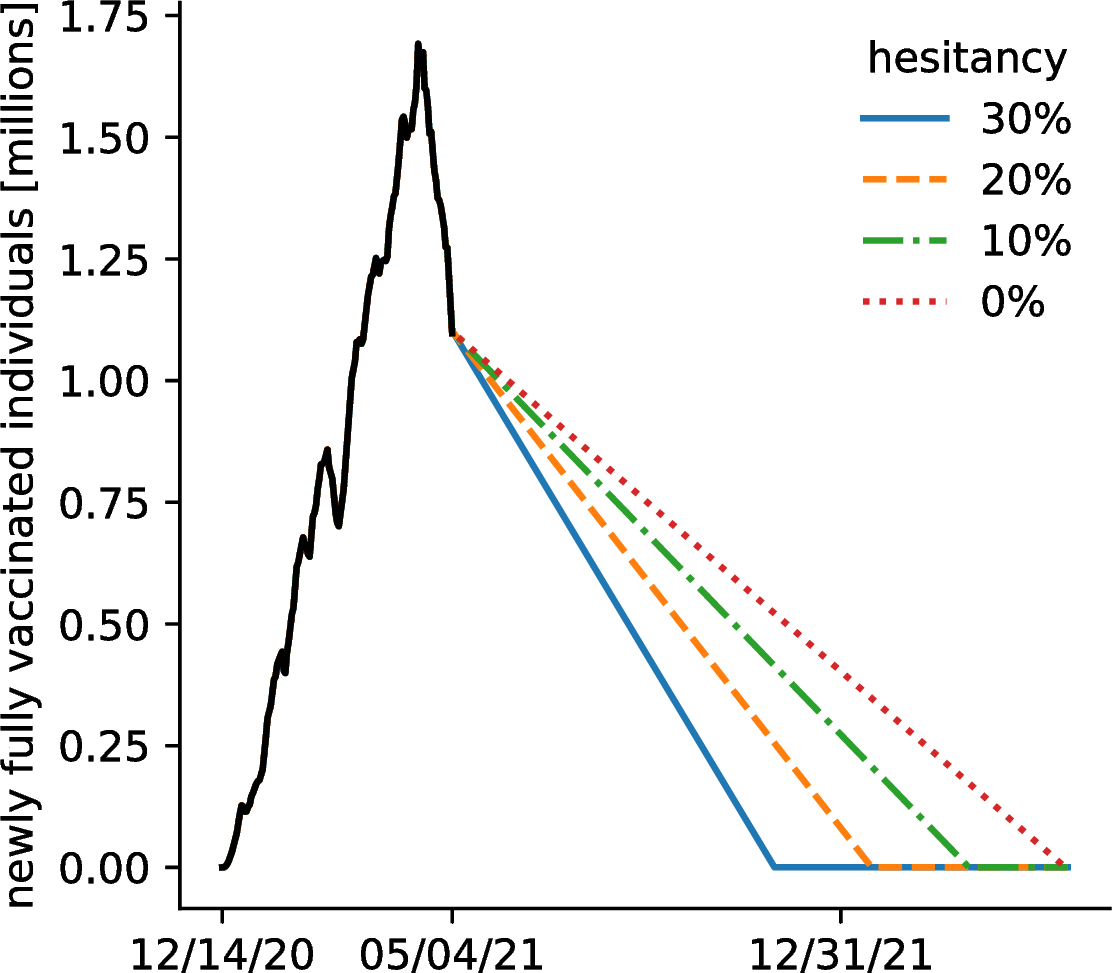

Supplement: S4 Fig — In the model, the number of newly fully vaccinated individuals each day is set to 50% of the 7-day average of the total number of administered doses (black line). Colored lines show predictions of the future speed of the vaccine roll-out for different levels of vaccine hesitancy. (TIF) [file pone.0259700.s004.tif]

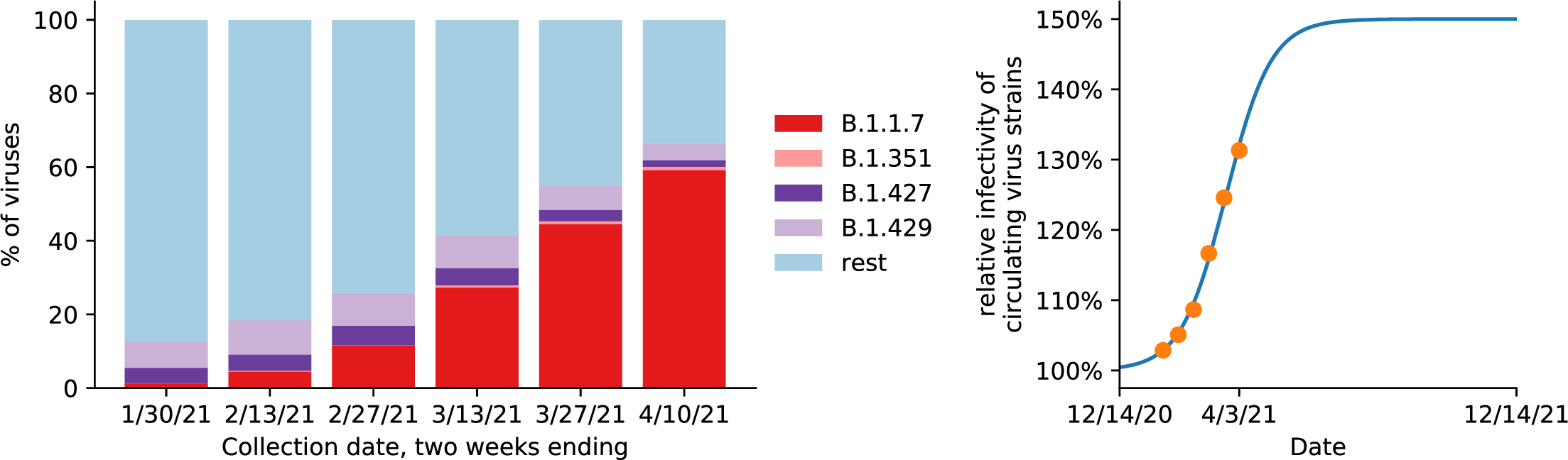

Supplement: S5 Fig — (A) Prevalence of several variants of concern based on > 40, 000 sequences collected through CDC’s national genomic surveillance since Dec 20, 2020 and grouped in 2-week intervals [42]. (B) For the midpoint of each two-week interval, the relative infectivity of circulating virus strains based on a 50% increased infectivity for B.1.1.7 and B.1.351 and 20% increased infectivity for B.1.427 and B.1.429 is shown (orange circles). A fitted logistic equation with asymptotes at 100% and 150% projects the future relative infectivity (blue line). (TIF) [file pone.0259700.s005.tif]

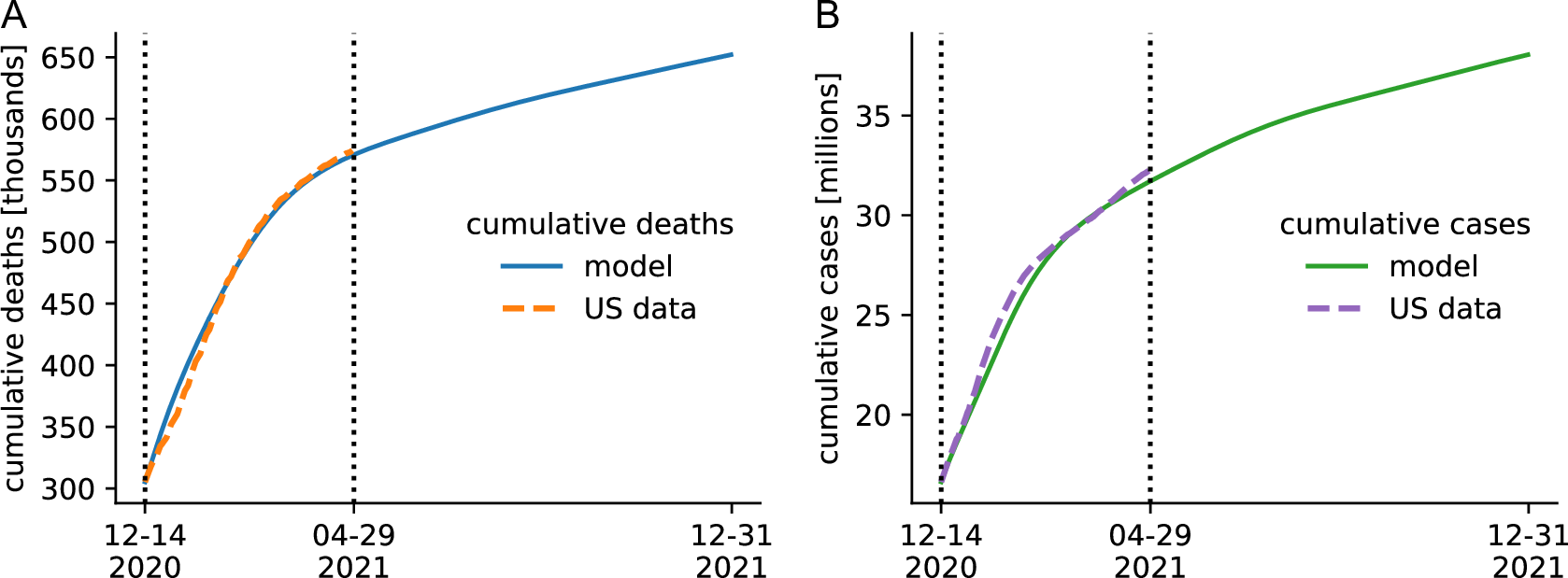

Supplement: S6 Fig — The observed (dashed line) and model-predicted (solid line) cumulative deaths (A) and cases (B) are shown. The model parameters used are described in Table 1 and in the first row of S1 Table. (TIF) [file pone.0259700.s006.tif]

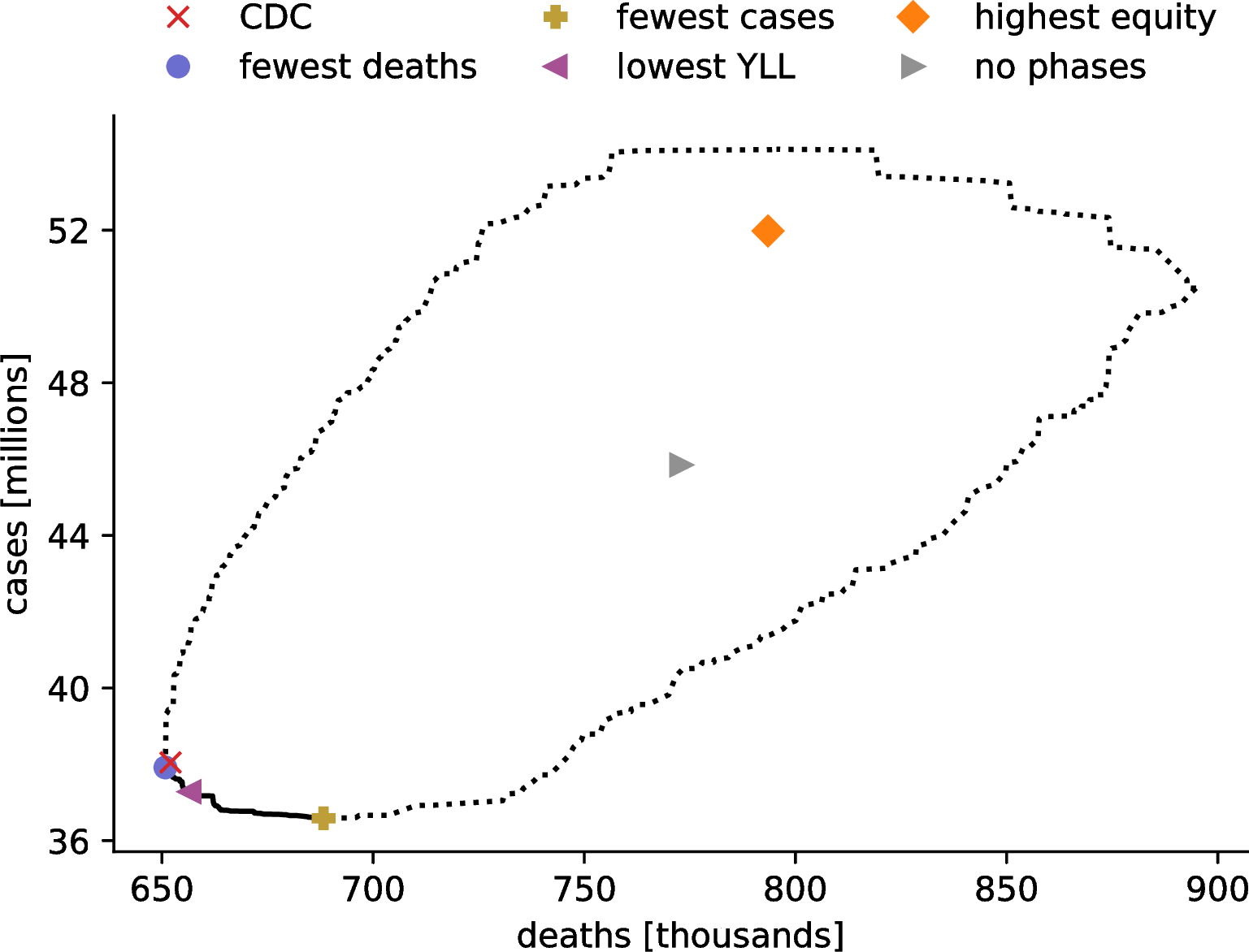

Supplement: S7 Fig — The death and case count of all 17.5 million evaluated meaningful vaccine allocation strategies fall within the dotted region. For strategies on the Pareto frontier (solid line), there exists no other strategy that performs better in one objective (minimizing deaths or cases) while not performing worse in the other objective. The death and case counts resulting from six specific allocations are highlighted. (TIF) [file pone.0259700.s007.tif]

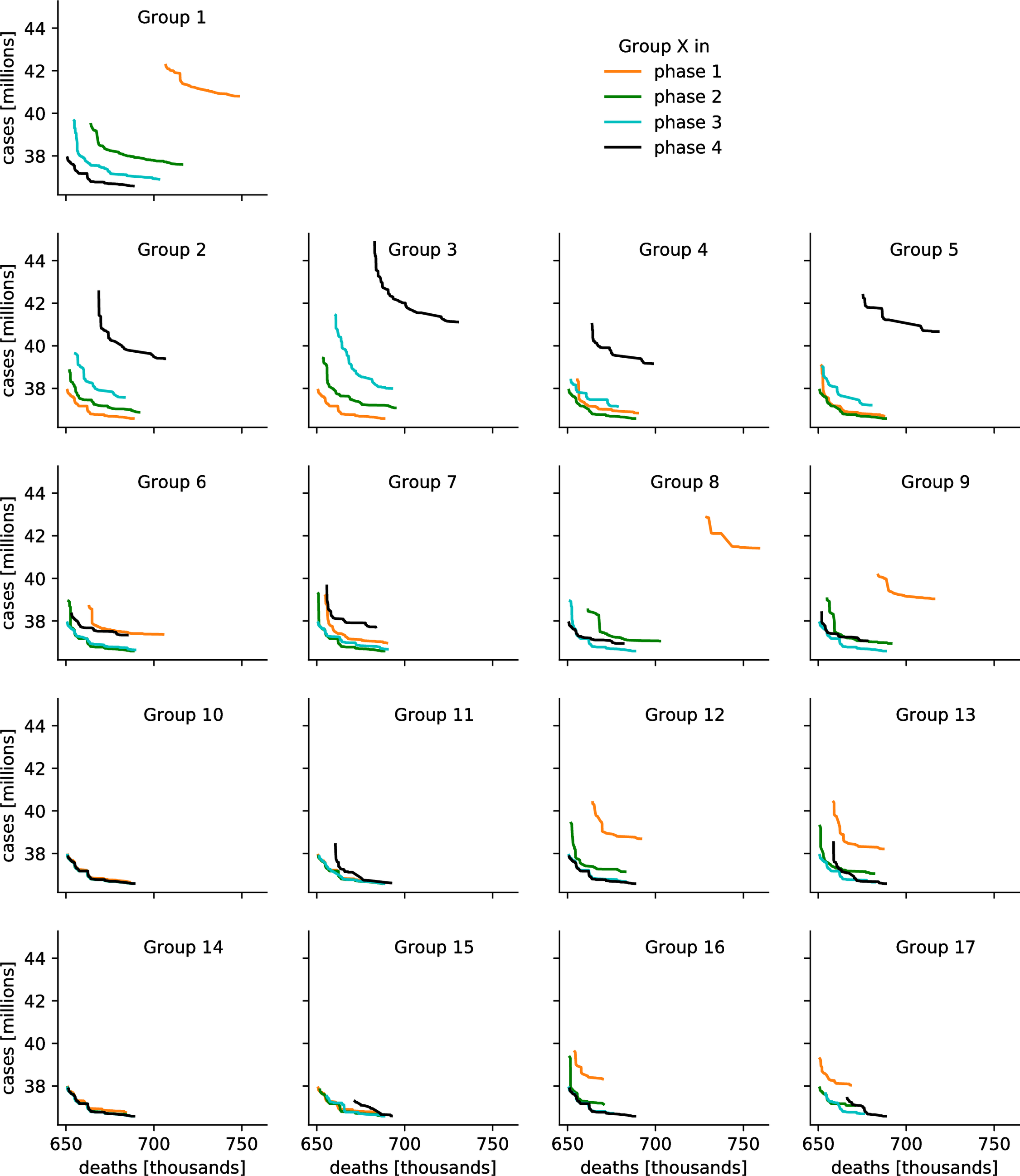

Supplement: S8 Fig — Each subpanel shows four Pareto frontiers. For each frontier, one sub-population’s priority phase is fixed (see Table 2 for group characteristics). For strategies on the Pareto frontier, there exists no other strategy that performs better in one objective (minimizing deaths or cases) while not performing worse in the other objective. (TIF) [file pone.0259700.s008.tif]

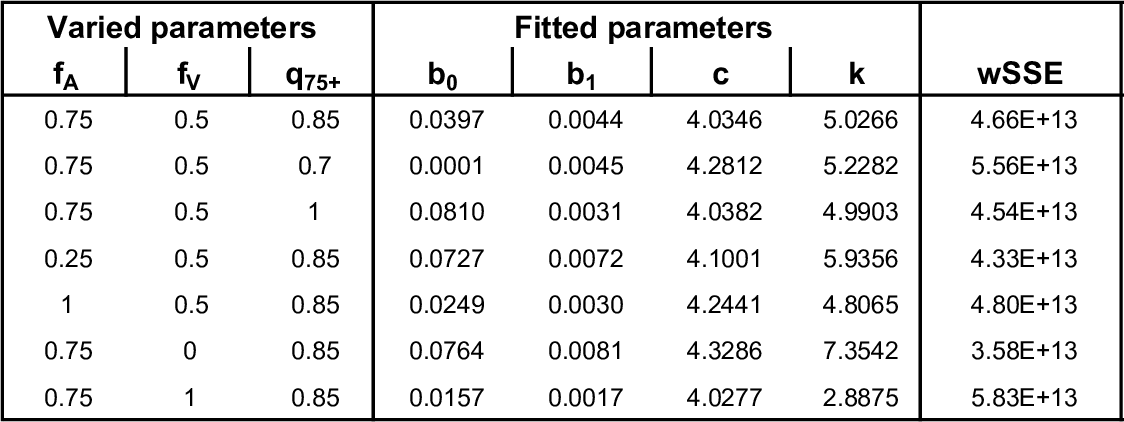

Supplement: S1 Table — For each scenario (described by the parameters in the three most left columns), 100 separate elitist genetic algorithms were performed and the parameters associated with the best fit are shown, in addition to the value of the cost function (wSSE) that the algorithm minimized. (TIF) [file pone.0259700.s009.tif]

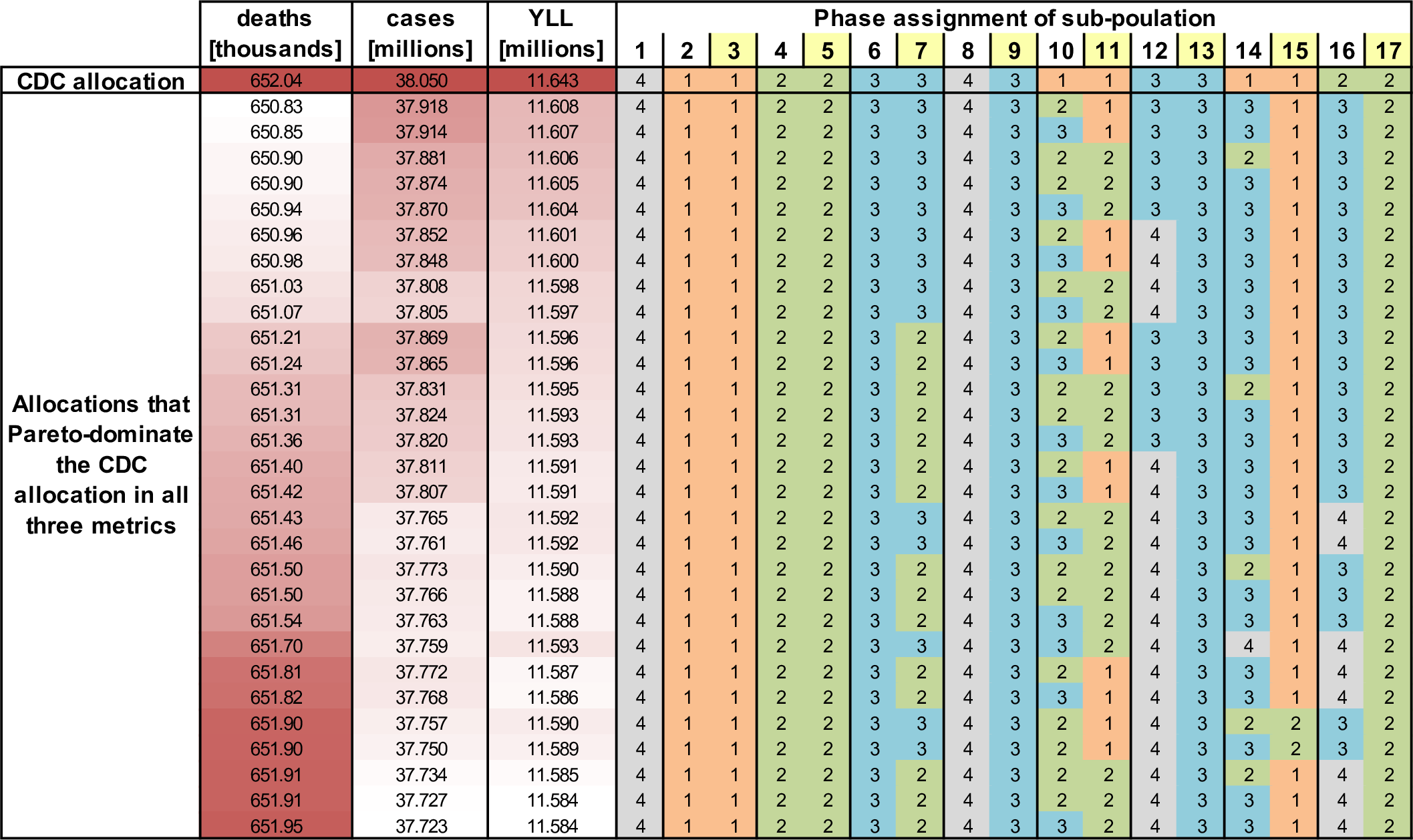

Supplement: S2 Table — This table shows the CDC allocation (first row) and all allocation strategies on the three-dimensional Pareto frontier that lead to fewer deaths, cases and YLL at the same time (bottom 28 rows). Sub-populations 1–17 are defined as in Table 2; sub-populations with comorbidities are highlighted in yellow. (TIF) [file pone.0259700.s010.tif]

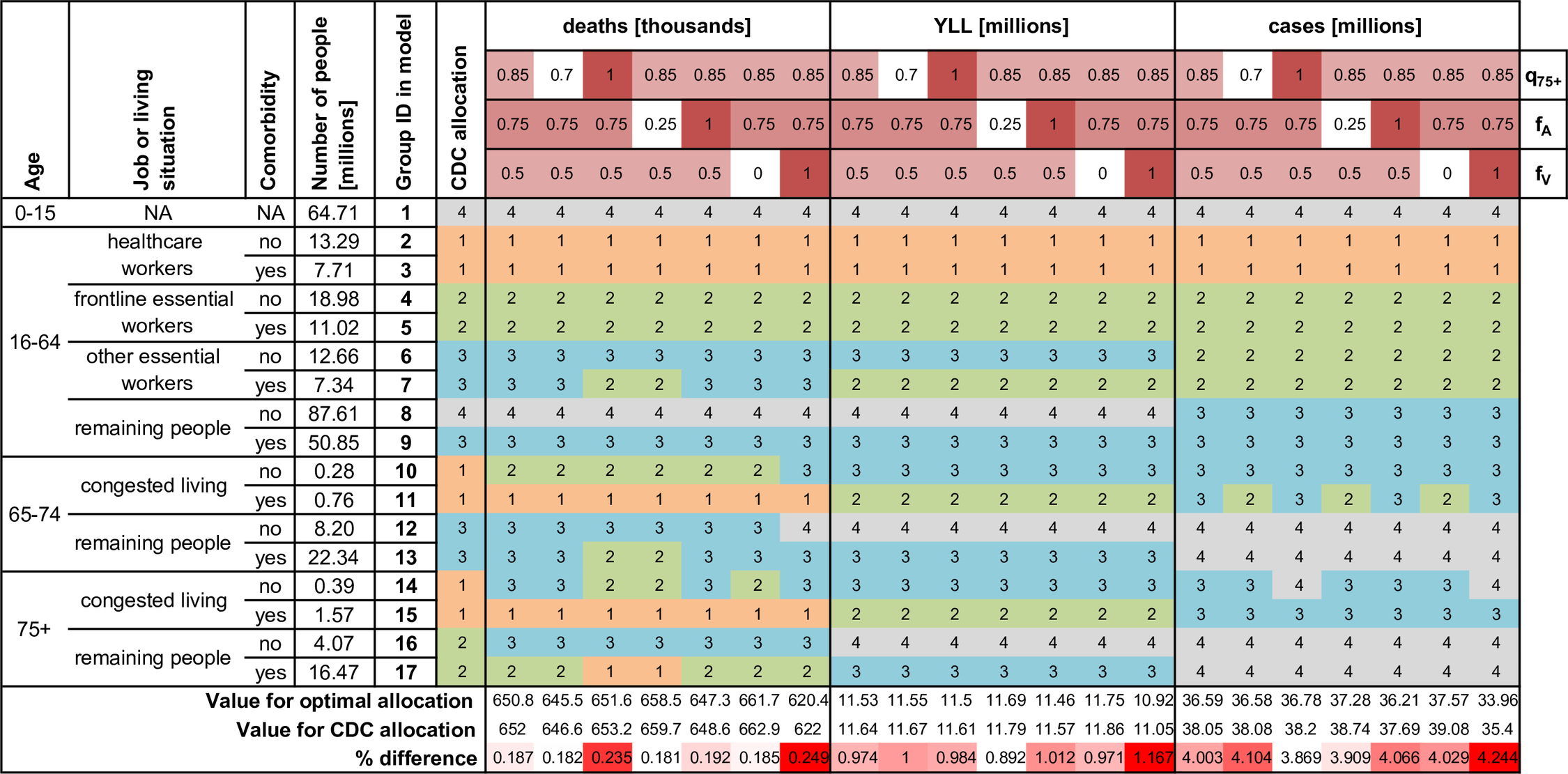

Supplement: S3 Table — For each sub-population (characteristics and population sizes defined in the left columns) and seven combinations of unknown disease parameters (q75+, the proportion of symptomatic infections among individuals 75 and older; fA, the relative contagiousness of asymptomatic infected individuals; fV, the relative contagiousness of vaccinated infected individuals), the priority phase corresponding to the optimal allocation strategy is shown. At the bottom, predicted outcomes (deaths, YLL and cases) resulting from the CDC allocation and the respective optimal allocation strategy are compared. (TIF) [file pone.0259700.s011.tif]

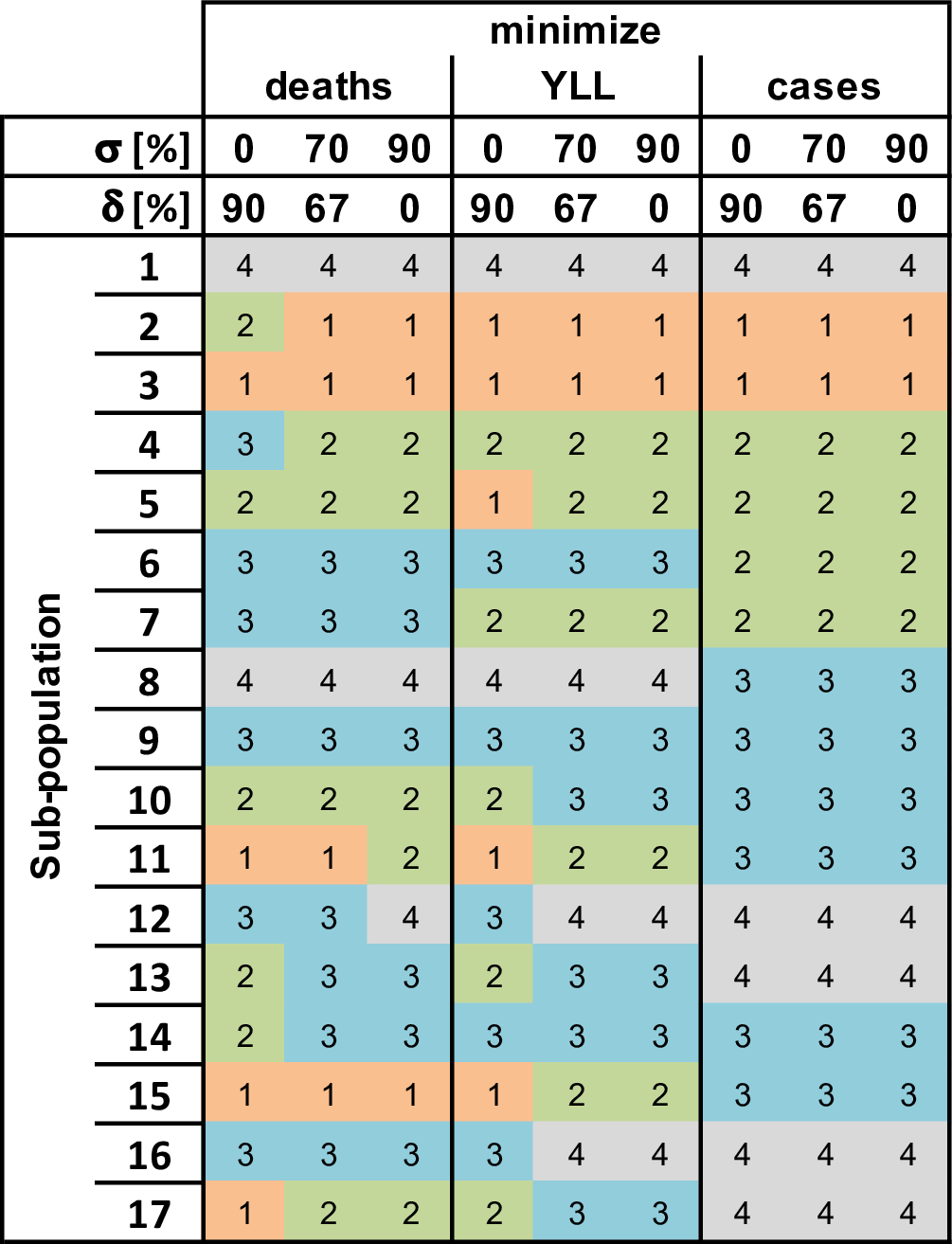

Supplement: S4 Table — For different types of vaccines with 90% effectiveness (specified by σ vs δ), the optimal vaccine allocation strategies with respect to three objectives (top row) are shown. Sub-populations 1–17 are defined as in Table 2. (TIF) [file pone.0259700.s012.tif]
